# Perspectives on cervical cancer screening among educated Muslim women in Dubai (the UAE): a qualitative study

**DOI:** 10.1186/s12905-015-0252-8

**Published:** 2015-10-24

**Authors:** Sarah Khan, Gillian Woolhead

**Affiliations:** College of Sustainability Sciences and Humanities, Zayed University, PO Box 19282, Dubai, United Arab Emirates; Department of Public Health and Policy, University of Liverpool, Liverpool, L69 3GL United Kingdom

**Keywords:** Cervical cancer, Screening, Pap smear, Muslim women, UAE, Qualitative study, Social constructivism, Interpretivism

## Abstract

**Background:**

Cervical cancer (CC) is the seventh leading cause of death among women in the United Arab Emirates (UAE), with most deaths attributed to late detection of this cancer. The UAE lacks a national CC screening programme. Thus, cervical screening is only performed opportunistically during women’s visits to health facilities. CC screening rates in the UAE are as low as 16.9 %, and little is known about the perspectives of the nation’s educated Muslim women regarding screening. Consequently, the aim of this study is to explore Muslim women’s perspectives towards cervical screening in Dubai to promote strategies for increasing its uptake, thereby leading to a decrease in morbidity and mortality associated with CC.

**Methods:**

Interpretivist and social constructivist epistemological approaches were applied for this qualitative study. Data were obtained through 13 in-depth interviews. Purposive and snowballing methods were used to recruit six South Asian women and seven Emirati women living in Dubai. Thematic content analysis was concurrently applied with comparative analysis to the data.

**Results:**

Four themes regarding women’s perceptions of CC emerged from the data. First, CC was considered a ‘silent disease’ that could be detected with early screening. However, it was also associated with extramarital sexual relations, which negatively influenced screening uptake. Second, women’s fear, pain and embarrassment, along with cultural influences, deterred them from undergoing screening. Third, a growing mistrust of allopathic medicine and impersonal healthcare promoted a negative view of screening. Last, women became aware of screening mainly when they were pregnant or receiving fertility treatment.

**Conclusions:**

The study highlighted a number of important factors relating to cultural, religious and sexual behaviour that shaped educated Muslim women’s perspectives on CC screening. Evidently, the current opportunistic approach to screening is flawed. A national awareness programme on CC screening should be developed, tailored to the sociocultural norms of the Muslim community, to promote knowledge regarding the causes of CC and the importance of screening.

## Background

Cervical cancer (CC) is the second most common cancer among women worldwide, with a mortality rate of 52 % [1]. In 2012 it accounted for 7.5 % of cancer-attributed female mortality and 266,000 deaths worldwide [2]. Moreover, mortality rates for cervical cancer are expected to increase by 25 % during the next decade, despite the fact that this is one of the most preventable cancers [3, 4]. By 2020, up to 0.7 million women could develop CC unless effective screening interventions are employed for early detection and to facilitate interventions for improving health outcomes [5]. In the United Arab Emirates (UAE), CC is the seventh leading cause of death among women [3], with most deaths attributed to delayed diagnosis of the disease [6]. In 2010, the rate of affliction with this cancer was 7.4 per 100,000 women in the UAE, revealing an increasing trend from previous years [7].

Cervical cancer is a slow-developing cancer of the cervix uteri of the female genital tract. Almost all cases are strongly associated with oncogenic infection with the Human papillomavirus (HPV) [1, 4]. While most infections resolve spontaneously, some may progress to cancer [1]. Symptoms remain elusive until the cancer progresses to advanced stages. Thus, seemingly healthy women need to be screened to detect asymptomatic precancerous lesions [1]. These lesions are 100 % treatable and their resolution can prevent CC from developing [3]. Early stages of the disease can be detected through the use of the Papanicolaou smear (Pap smear), which has a sensitivity range of 40–70 % [8]. Despite having a 15–25 % negative rate [8], this test is important for detecting dysplastic cervical cells.

In countries lacking national cervical screening programmes, the prevalence of CC is up to 75 % higher, with higher associated mortality rates, compared with countries that do have national screening programmes [4]. The UAE lacks a national CC screening programme. Thus, cervical screening is only performed opportunistically on women who visit health facilities, leading to screening rates being as low as 16.9 % [9, 10]. This situation represents a public health problem. Consequently, doctors in the UAE have emphasised the need for a nationwide screening programme to replace the current individualised screening providers [11].

Studies exploring perceptions of CC screening among Muslim women have drawn attention to various factors that are significant. Religion and culture, along with awareness of the signs and symptoms of this cancer, were found to be strongly associated with the uptake of screening [12]. A qualitative study conducted in Malaysia found that because Muslim women perceived CC as fatal, attempts at diagnosis were viewed as futile [13]. In a community-based study conducted on a diverse sample of 254 Muslim women in Chicago, many regarded affliction with this disease as a punishment from God and resigned themselves to fate rather than opting to take preventive action [14]. By contrast, another study in which American Arab immigrant women completed self-administered questionnaires revealed that faith in God removed the fear of cancer diagnosis in some Muslim women [15].

Quantitative studies on knowledge, attitudes and practices relating to CC screening were conducted on 299 Muslim women attending 16 randomly selected polyclinics within four governorates in Kuwait [8] and 500 randomly selected women visiting primary healthcare centres in Qatar [16]. These studies suggested that most women were unaware of CC symptoms or of the need to be screened. Other studies among Muslim women in Malaysia and Saudi Arabia [13, 17] have also revealed this lack of awareness. Moreover, it was found that women often assumed that they were healthy because they were asymptomatic and, therefore, did not consider screening beneficial [15].

Conversely, in the UAE, a quantitative study on CC screening conducted on 350 Muslim schoolteachers, all nationals of the Emirate of Sharjah, showed that although 84 % were knowledgeable regarding CC and screening, most of the women had never had a Pap smear [18]. Moreover, the study found that 42 % of the women believed screening would be painful, 36.1 % were embarrassed and 17 % perceived themselves as healthy and, therefore, not in need of screening. Only 5.5 % refused a Pap smear because their husbands did not give them permission to undergo the test. Other studies have also found that pain, embarrassment, fear of the test and lack of referrals for screening, by healthcare providers were barriers to screening uptake [8, 15, 16]. Several studies have emphasised a preference for being attended by women physicians [8, 16, 19, 20]. One qualitative study, that explored the attitudes of 28 Muslim women in England towards self-sampling for HPV also indicated that the presence of male physicians was a barrier to screening [21].

Cervical cancer and screening in the UAE is an underexplored subject. To the best of our knowledge, no studies in this area have been conducted in Dubai, and there have been no qualitative studies to date on CC screening within the UAE. Most studies that have explored knowledge, attitudes and practices of Muslim women regarding CC and screening have used a quantitative approach. However, only qualitative studies can reveal the complexity of influences that translate into human perceptions [22, 23]. This study seeks to fill this knowledge gap to understand the perspectives of immigrant and native Muslim women in the UAE and to explore possible influences of acculturation, religion and ethnicity on CC screening. Information in these areas is vital for planning effective CC screening programmes for Muslim women in the UAE.

## Methods

We used social constructivist epistemology and an interpretivist approach to acquire an understanding of the different perspectives held by individuals that reflect a subjective, socially constructed reality [24]. Social constructivism assumes that reality is subjective and comprehended through the social, historical and political experiences of a society. An interpretivist approach seeks to understand rather than to simply identify human interpretations of experiences and behaviours [24]. Social constructivism enables reality to be constructed through interpretation and accepts that people can be different despite their “sameness” [22]. Although women are biologically similar, perceptions of disease susceptibility, severity and attitudes towards interventions are shaped through their personal, cultural and social beliefs [22, 25]. Thus, human behaviour is complex and fluid and requires a more subjective approach to be better understood.

### Setting

We conducted a qualitative study using in-depth interviews to gain a rich understanding of Muslim women’s perspectives towards CC screening [26]. The UAE is located within the Arabian Peninsula and has a population of 8,264,070 of which 16.6 % are Emiratis and 83.4 % are expatriates [27]. South Asians comprise 42.3 % of the expatriate population, with the remainder comprising residents of other Arab countries (23 %), other Asian countries (12.1 %) and other regions of the world (6 %) [27]. The UAE has a high per capita income with a gross domestic product of 4 %. It ranks 41^st^ in the Human Development Index [27]. Of the total population, Muslims comprise 96 % [16, 27–30].

Dubai is the largest city and the second largest emirate of the UAE [28]. The estimated population of Dubai in 2009 was 1,722,000, of which 77 % were men [30]. Islam is Dubai’s official religion. Based on the findings of a survey conducted in 2002, roughly 60 % of Dubai’s population is Muslim, 14.72 % of the population is Christian, and the remaining 26.12 % follow other religions [31].

### Participants and recruitment

As every individual can potentially express a large number of perspectives, a small sample was sufficient to elicit rich data [32]. Initially, participants were selected through purposeful sampling to ensure the acquisition of required data [26]. Muslim women residing in Dubai who were at least 18 years of age, married, had some formal education and were aware of CC screening were included in the sample. Conversely, women below 18 years of age, unmarried, with no formal education and who were unacquainted with Pap smears were excluded from the sample. Given the sensitive nature of the study, and the need to respect the norms of modesty in a Muslim country, unmarried and uneducated women aged below 18 years were excluded. The principal researcher emailed an information sheet to potential participants at her workplace, and to acquaintances, requesting them to indicate their willingness to participate by contacting the principal researcher. Of the 20 women contacted, four agreed to participate. Further participants were recruited through the use of snowball sampling techniques. Thus, participants and women who were initially contacted introduced the researcher to other women willing to be interviewed for the study (see Table 1) [26, 33]. Once the women had demonstrated their willingness to participate in the study, the researcher spoke with them in person or via the phone to ensure that they met the recruitment criteria. Seven Emirati women and six South Asian expatriate women were recruited for the study.

**Table 1:** Interview and recruitment details

Interview	Nationality	Duration	Location	Recruitment
1	Expatriate 1 (Ex 1)	52 minutes	At the participant’s house	An ex colleague from the researcher’s workplace
2	Emirati 1 (Em 1)	1 hour 7 minutes	In the researcher’s private office	An ex colleague from the researcher’s workplace
3	Emirati 2 (Em 2)	1 hour	In the researcher’s private office	Recruited through an administrative assistant in another department at the researcher’s workplace
4	Emirati 3 (Em 3)	51 minutes	In the researcher’s private office	Recruited through the administrative assistant in another department at the researcher’s workplace
5	Emirati 4 (Em 4)	52 minutes	In a conference room at the participant’s workplace	Recruited through an administrative assistant in another department at the researcher’s workplace
6	Expatriate 2 (Ex 2)	40 minutes	In the participant’s office.	An ex colleague from the researcher’s workplace
7	Expatriate 3 (Ex 3)	1 hour 5 minutes	Skype interview from the participant’s house	Recruited through a participant
8	Expatriate 4 (Ex 4)	53 minutes	In a coffee shop	Recruited through a participant
9	Emirati 5 (Em 5)	56 minutes	In the researcher’s private office	Recruited through an administrative assistant in another department at the researcher’s workplace
10	Expatriate 5 (Ex 5)	52 minutes	At the participant’s house	Recruited through a participant
11	Expatriate 6 (Ex 6)	54 minutes	At the participant’s house	The researcher’s acquaintance
12	Emirati 6 (Em 6)	48 minutes	In the participant’s private office	Recruited through a colleague at the researcher’s workplace
13	Emirati 7 (Em 7)	53 minutes	In the participant’s private office	Recruited through a participant

### Data collection

Data were obtained from 13 in-depth semi-structured interviews. An interview guide was developed using open-ended questions relating to participants’ awareness of CC screening, perceived susceptibility to CC and its severity, as well as barriers and motivating factors related to screening. Two pilot interviews, one with a national and one with an expatriate, were conducted to identify the weaknesses and limitations of the interview guide [34]. The duration of interviews ranged from approximately 40 minutes to 1 hour. Initial questions were aimed at gradually easing into the conversation. Some questions were subsequently repeated to enable participants to elaborate on their initial answers [35]. Interviews were held in a venue of the participant’s choice to facilitate free discussion on a potentially sensitive subject. Some participants chose to be interviewed at their own homes, while a few preferred to use their workplace as the interview venue. Several interviews were held at the principal researcher’s private office, one was held in a coffee shop and one was conducted over Skype [36]. Voice recorders were used to record the interviews and field notes were taken to document nonverbal communication and interruptions. Interviews were transcribed *verbatim* using the Microsoft Word document application with the aid of a transcribing software, ‘Transcribe’.

Ethical approvals were obtained from the Zayed University and the University of Liverpool, owing to the respective affiliation of the researchers to these institutions. Participants were provided a participant information sheet to read before the consent forms were signed. The participant information sheet explicitly stated that findings from the study would be published. Written consent was obtained prior to conducting the interviews, and participants were assured that they could withdraw or refuse to answer questions at any time. Sensitive questions relating to personal sexual activities were avoided to respect the modesty norms of Muslim women. However, most women voluntarily offered their views on sexual behaviour within the community during the course of the interviews. Interviews were transcribed and coded by the researchers, and numbers were used instead of names to ensure confidentiality and anonymity. All identifying traits in interviews that could be traced back to participants were removed during transcription. Because women disclosed information that could be considered sensitive in their society, it was essential to protect their identities.

### Data analysis

Interviews were transcribed *verbatim* as Word documents. The entire transcript was colour coded and labelled according to the information relayed. Similar codes were grouped into themes and subthemes. Thematic content analysis was applied to identify emerging and recurrent themes, and comparisons were carried out between and within the groups of participants [25].

### Methodological considerations

Each participant’s responses generated a range of subthemes and themes that later exhibited saturation. Thus, a larger sample was not required [32].

The principal researcher’s background as an educated Muslim and a married woman residing in Dubai was similar to that of the participants. The second researcher did not have any direct contact with the participants and was involved in aspects of planning, data analysis and authoring the research. Participants were aware of the researcher’s position and felt comfortable enough to bring up perspectives on sexual relations on their own. The principle researcher was in the unique position of simultaneously being an insider, with shared background similarities with the participants, and an outsider as a result of having medical training. This enabled an exploration of perspectives from different positions. The researcher made a conscious attempt to avoid biased and leading questions to prevent the emergence of any preconceptions [37, 38].

Rigour was maintained throughout the study by employing several strategies [39]. These included: prolonged engagement with participants during interviews, thick description and supporting quotes representing all participants that gave credibility to the acquired information [23, 40]. Despite being small, the sample exhibited a saturation of views [25]. Moreover, participants had an opportunity to reconsider their responses when the researcher reminded them of their previous answers [41].

The researchers attempted to use consensus obtained in relation to some of the results to show agreement and deviance of the participants’ views. While frequencies are not well accepted within qualitative research, counts were included in some cases to reflect on the significance of a theme for participants [39, 42]. All of the steps conducted for this study have been clearly described in this paper, including the study setting and procedures applied for recruiting participants, as well as collecting and analysing data. This enhances transparency and enables the transferability of the study within different settings for other researchers to derive their own inferences [41]. The researchers performed repetitive checks and peer debriefing throughout the study to include consideration of alternative interpretations [23, 39].

## Results

The four main themes emerging from the study were:

- ▪ perspectives on CC and their influence on screening uptake;
- ▪ perspectives on CC screening and their influence on screening uptake;
- ▪ other factors influencing CC screening uptake; and
- ▪ current awareness regarding CC screening and future needs.

Similarities and differences within and between the two groups of women, namely nationals and expatriates, were considered. In the participant codes, “Em” was used to denote Emiratis and “Ex” was used for expatriate women. The key findings of the study are summarised in Table 2.

**Table 2:** Summary of key findings

Perspectives on cervical cancer and its influence on screening uptake
● CC was considered a ‘silent’, curable disease with a precancerous stage, which was detectable early through screening. Despite this knowledge two expatriates had never been screened due to lack of perceived risk.
● CC was associated with sexual relations and promiscuity, which had a negative impact on screening. Most participants believed CC was caused by poor hygiene.
● ‘Evil eye’ could be responsible for causing CC but did not prevent women from seeking medical help.
Perspectives on cervical cancer screening and its influence on screening uptake
● CC screening was considered a routine procedure that was uncomfortable, embarrassing and possibly painful, which discouraged screening, in some cases.
● Religion did not deter women from being screened, however cultural norms could dissuade women from being screened.
Other factors influencing cervical cancer screening uptake
● Most women preferred being screened by female doctors, however nationality and religion of the doctor was not a major consideration.
● All women preferred experienced, friendly doctors with whom they could communicate with in their preferred language.
● There was growing distrust of allopathic medicine, which had a negative influence on CC screening.
● Without health insurance, CC screening would be not be a priority for most women
Awareness of cervical cancer, screening and future needs
● Awareness of CC and screening was lacking in Dubai; gynaecologists, friends and family were the commonest sources of information.
● All women would surf the internet for information on CC
● General agreement was that awareness programmes should target the younger generation in schools and universities. Some participants felt schoolgirls were too young to be exposed to CC awareness.
● The government has an important role in increasing cervical screening uptake, possibly through pre marital screening and education.

### Perspectives on cervical cancer and their influence on screening uptake

All seven Emirati participants had been screened for CC after their children were delivered or as part of their treatment for infertility after their marriages. Prior to these medical interventions, they were unaware of healthcare pertaining to CC.

*I had actually no knowledge of…anything [emphasis on any] related to…that female part that…organ until I actually conceived…and then when I had to go and see a gynaecologist…until…basically after delivery…when my gynaecologist insisted that I get a Pap smear.* Em 2

Two expatriates were screened for CC after undergoing their deliveries in other countries, two were screened in Dubai, two had never been screened and one had only been screened once, 15 years ago, while undergoing treatment for infertility-related issues. Expatriates were as unaware of CC screening as Emirati women prior to consulting gynaecologists during pregnancy and for fertility-related issues. Two expatriates whose deliveries took place outside of the UAE remained unaware of screening even after their children were born.

#### The nature of the disease

Both Emirati and expatriate women believed that it was possible to have CC without being aware of it, with symptoms becoming obvious only after reaching advanced stages.

*…because it doesn't have any signs… it is silent…[a] very silent and quiet illness…in quiet illness…it shows suddenly and when it reaches a very bad level.* Em 5

A common issue that participants raised, while expressing agreement regarding the importance of screening, was the need for early detection of a “silent” disease to enable successful treatment. This was perceived as important even though the test was not considered as a preventative:

*It depends on which stage it will be diagnosed. It is the same like for each kind of cancer…if it is in the beginning and they could spot it, I think it will be easy [to cure] by removing something, the uterus or whatever…the doctor will decide about that…but if it is in late stages, same like my relatives…I think it will not be curable.* Em 7

Despite having prior knowledge of the existence of screening for CC, two expatriate women had never been screened because they had not experienced any symptoms of CC. One of the women questioned the usefulness of the test:

*You cannot get every single organ of your body checked for cancers…Since this test is not going to prevent it, the only thing it can set off is true or false alarms…so I am just not willing to put myself through it.* Ex 3

Only two Emiratis and one expatriate knew that CC was preceded by a precancerous stage that was detectable through CC screening. Both groups were uncertain about the signs of CC and speculated that there could be abnormal bleeding, cessation of periods, discomfort, pain, itching and irritation, a lump in the uterus and loss of bowel control.

*I guess…bleeding could be [there]…when you have something…in your uterus…[a] lump or something…could be… but…not more. But it could be bleeding…um, irregular period…and… as I said [a] lump in the uterus…like that…uh forgive my ignorance in this subject.* Em 7

All of the women understood that CC was a progressive disease that could spread to other organs of the body. Three participants considered CC fatal, but this did not affect their views on screening.

#### Likelihood of getting cervical cancer

Most participants believed that promiscuity increased the risk for CC. However, there were conflicting views regarding polygamous marriages. Islam allows men to have four wives at one time, with each wife being legally married to the man. While participants believed CC was linked to sexual relations, they did not feel that being in a polygamous marriage increased the risk for CC.

*It help[s] us [allowing polygamous marriage]…it should help us…because [in] Islam if you learn good Islam…or right Islam…it is about taking care of women, about having a good relation with only one man.* Em 5

Only two expatriates suggested that polygamous marriage *increased* the risk for CC.

*…because you have four partners and even [if] one of those four partners have it…and then for the women…because if the man is transmitting that infection, yes, then all four women could get it.* Ex 3

The participants generally considered that Muslim communities were more cautious regarding engagement in extramarital relations. However, promiscuity, which was considered a risk for CC, was not regarded as being entirely absent. A common view was that promiscuity in men was overlooked, while women were held to higher standards of morality.

*I think women have more [of] the fear of God in them…you know, basically not to be maligned…not to be blamed…because if a woman is caught, the woman…is the one to blame…[while] the man goes scot-free.* Ex 5

The women’s views on marriage and promiscuity had an impact on screening uptake as they felt obliged to portray their marriages as being successful. Therefore, to avoid the embarrassment and stigma of their husband’s infidelity, they would avoid screening.

*It’s like we try to protect the image of a stable marriage…faithful spouse…and to suggest otherwise is to suggest your spouse could have [transmitted], you know, STD [sexually transmitted disease] to you would also basically…tell people that he [has] been unfaithful to you…which is not something you want other people to know…We always tend to feel like we bear the stigma.* Em 2

The notion of CC being infectious and not necessarily related to sexual behaviour was common to both groups of women. This was evident from repeated references to poor hygiene and the use of public toilets as a cause of CC.

*It can be caused by some kind of virus…so it is a…if you are using a public toilet…or something, you could catch it.* Ex 3

Another common belief, particularly among Emirati women, was that *hasad*, or the “evil eye”, could cause CC. *Hasad* means envy or people wishing ill to others who possess what they desire but lack. This ill will could manifest as a disease, including CC. Expatriates also expressed a belief in *hasad*, but seemed more inclined to dismiss it as a cause of CC.

*They feel that we get the cancer because…people look at us…They give us hasad…Because [for] most of the cancer[s] they think the reason is hasad....Because they didn’t say Mashallah [God has willed it].* Em 3

*I do believe in hasad and evil eye, but diseases are not because of that.* Ex 2

This belief did not have an impact on screening as most participants agreed that both religious and medical cures would be sought for diseases even if they were attributed to the “evil eye.”

*…[the] doctor helps…but [the] Quran [does] 90 %…Allah’s word is different than doctors…but [the] doctor helps…I don’t say [the] doctor [does] nothing.* Em 4

### Perspectives on cervical cancer screening and their influence on screening uptake

The focus of this theme was perspectives on and experiences of screening among Muslim women in the UAE and their influence on screening uptake.

#### Screening experience

Most women described the procedure as uncomfortable, with three women reporting that it was painful. Those who experienced pain were fearful of being screened again.

*The scraping out…wasn’t painful. It was a ticklish, uncomfortable feeling. That is about it.* Ex 6

*I found the Pap smear very* [emphasised] *uncomfortable, very* [emphasised] *painful, and this is why I didn't want to go through it again…Some people say it is not [painful]…it is just discomfort…but for me it was pain.* Em 2

Embarrassment was a common concern among expatriates. Conversely, only one Emirati woman indicated self-conciousness regarding being screened. This finding indicates possible cultural differences between the two groups despite sharing a common religion.

#### Religion and culture

A common view was that Islam strongly encouraged women to take care of their health, even if this meant exposing their private areas. Women believed that even ordinarily prohibited acts such as consuming alcohol were allowed by Islam to preserve life. Rather than religion, cultural norms, such as subservience and modesty on the part of women, explained their hesitation to undergo screening. However, religion did not have a negative influence on CC screening uptake.

*I am sure about it…100 % this is halal [permitted]*. Em 3

*…for medication purposes, you are even allowed to take cough syrup that has alcohol…so, if such things Allah has allowed, then He is definitely not going to penalise you for this.* Ex 6

Culturally, the women in this study placed the needs and expectations of their families and husbands before their own. This influenced CC screening uptake.

*We always feel that we need to do everything for everyone…we always take the back seat…we need to look after our children…we need to do things for our children…we need to do things for our husbands.* Ex 5

Three Emirati women and two expatriate women preferred to obtain the approval of their husbands for screening. Four Emirati women indicated a preference or expectation of being accompanied when going for CC screening. Not having someone to accompany them could negatively influence screening uptake.

*Because of the culture…because of the wrong thoughts…that is it…They always feel like you have to have a bodyguard…always to keep people away from you…but this…doesn't help…Who wants to do anything will do it…yeah.* Em 5

### Other factors influencing cervical cancer screening uptake

This theme encompassed various other factors that influenced CC screening uptake.

#### Doctors and their influence

Ten women indicated a clear preference for attendance by female doctors, stating personal comfort and religion as reasons for their preference.

*I feel shy…[of a] male…and if…[a female doctor is] available…why should I go to [a] male?* Em 4

*I would definitely prefer a lady…[as the] number one [choice] because [according to] Islam, we are supposed to look for a lady doctor first. If it is something that requires exposing our bodies…we should try and find a lady doctor. If not, you can always go to a male doctor…medically, it is allowed…My second reason is my own comfort.* Ex 3

One Emirati woman did not believe that gender mattered, while another Emirati preferred male doctors, because she considered them to have superior skills to female doctors.

*…but I think, in my mind, [a] man is better than [a] woman…the doctor…I think [he] [has] more experience and…he has different thinking from a woman.* Em 3

The doctor’s religion was not a major consideration. Only two women indicated a preference for a Muslim doctor.

*No…no[t] important…Muslim, non-Muslim…we are all human…Muslim, non-Muslim is for himself.* Em 4

An Emirati woman expressed a preference for Arab doctors. Conversely, one Emirati and three expatriate women discredited Arab doctors. Among the latter, whereas the Emirati woman felt embarrassed about being examined by someone of her own nationality, the expatriates felt that Arab doctors were unqualified.

*I would prefer going to an Asian doctor…but definitely not [to] some Middle Eastern doctor…honestly…not someone from here who is educated here…or maybe from the Gulf countries…I wouldn't want to risk myself .* Ex 2

All of the women felt that it was essential that the doctor spoke a language they understood. At times, a native language was preferred for precise expression.

*Of course, Arabic will help me more…will help me to say anything I want to say, because sometimes you try to find the…words in English…you cannot find it…but…in all cases you can communicate with the doctor.* Em 5

All of the women preferred to visit experienced doctors who also made them feel comfortable. A patronising attitude on the part of a doctor would discourage participants from returning and deter them from undergoing screening regularly.

*Yes…so [there] is…this whole condescending way of speaking to…you know, patients who you know…probably did do something…culturally [or] religiously unacceptable…It’s just…putting them off the idea of seeing gynaecologists.* Em 2

Three expatriates and three Emirati women mentioned mistrust of allopathic medicine, translating into increased reservations about the need for CC screening. This wariness of allopathic medicine led them to adopt alternative medicines such as homeopathy, religious cures as well as herbal treatments.

*It is…it’s thought…you know, just…all [of] this dua [prayer]…and spiritual cures…and hakims [Islamic healer]…and then homeopathy…All are considered to be better than allopathic treatments.* Ex 4

One expatriate and one Emirati felt that healthcare services had become impersonal and business-like. One expatriate also questioned the transparency of medical research.

*No…[oh] my gosh…[the]medical system is really bad here…because it is all to do with business.* Ex 6

#### Facilities and insurance for Pap smears

Satisfaction with medical facilities was evident and the quality of facilities did not, therefore, have a negative impact on screening uptake.

*We have everything, clean hospitals, we have labs for the results…when we go to a consultation room…[for] doctor consultation, you will find [the] best beds…uh the ultrasound system, for example, [is] one of the best.* Em 6

All participants had health insurance. However, one expatriate and two Emiratis stated that their insurance did not cover Pap smears or visits to their preferred doctors.

*No…no, this clinic, they do not take insurance…but I don’t think the insurance covers the test…I paid 1100 [dirhams] [approximately US$ 300].* Em 6

Those without insurance would most likely not be screened. Even if they could afford the smear, their priorities lay elsewhere.

*No…because [the] woman she is [a] mother. She will keep the money for her children. If she is not a mother, she will [also] not… put [aside] money to [do a] test…She will go buy makeup or perfume here in [the] UAE. It is like this.* Em 5

### Current awareness of cervical cancer, screening and future needs

This theme covered perspectives on women’s current awareness levels regarding CC and screening and suggested avenues for improving uptake of CC screening.

#### Current awareness

Only one Emirati had heard of CC-related awareness campaigns. All of the other participants mentioned a lack of initiatives and awareness of CC.

*We are not an aware community…Yes, see, the lifestyle we have…we are second or third [for] obesity and diabetes…This is not an aware community about what is happening.* Em 6

Despite the general dearth of initiatives in this area, some participants pointed to comprehensive insurance plans, posters and leaflets on CC screening seen in certain clinics and hospitals. Apart from gynaecologists, the most common source of information in this area was friends and family. All of the women affirmed they would refer to the internet for more information on CC and screening.

#### How to increase awareness and improve CC screening uptake

All of the participants agreed that CC screening awareness was needed. Moreover, all except one Emirati recommended targeting the younger generation rather than the older generation. The most commonly suggested platforms to increase awareness were schools and universities.

*The best way to do it is starting with school. I mean school and university. This is the only way, because when it comes a curriculum, nobody can question it…because there is a saying in Arabic 'la haya fil ilm’[meaning] there is no shame when it comes to education.* Em 1

However, some participants were concerned that awareness of CC at the school level meant exposing girls to knowledge of the disease at a very young age before they were married. This hesitancy to educate unmarried girls on a disease associated with sexual behaviours reflects cultural norms.

*School, I don’t think so…I think school doesn’t even know about this [cervical cancer] also…maybe university, yes… because females, a lot of them are married. Half of them are married…Maybe they will need [to know], but in school they are still babies, they are young.* Em 4

There was general agreement that the government could help to improve the uptake of CC screening by reducing the cost, carrying out awareness campaigns and sending reminders to women.

*The government has to go search [for] and take people…to take the test…go to clinics and hospitals and tell people.* Em 4

Premarital testing was considered a potential avenue for promoting CC screening. People getting married in the UAE undergo a premarital health check up to screen for genetic diseases.

*For marriage…um…we have here checking for diseases [of] blood, for anaemia…you do [this] in the UAE…and [for] other family diseases.* Em 3

One Emirati mentioned that couples receiving the government marriage fund were expected to attend six educational sessions before they got married. This was suggested as a suitable venue for promoting awareness of sexually transmitted diseases and CC.

*We have like [a] marriage fund…they do lectures…six lectures that both male[s] and female[s] must attend before marriage…If it [were] in my hand[s]…I [would] add two lectures only about health…what health problem[s] might come from marriage.* Em 6

One Emirati and two expatriates expressed the view that they would be more accepting of CC screening if there were alternative screening tests available that did not require exposure of their private parts.

*Oh my God…my gosh, it is so awkward…If [only] there was…another way of doing it, it would be better…They [would] be much happier to take it…since it [would be] just a blood test and not something where they [would] have to take off all their clothes.* Ex 6

Given the widespread use of media, social networking sites, TV and radio were considered important and efficient ways to increase the uptake of CC screening.

*They could use this networking, social networking, social medias to raise awareness because everybody would go through it these days…right… and it spreads words like anything…much faster than any other media.* Ex 1

An Emirati suggested wider outreach would be possible if all doctors, and not only gynaecologists, shouldered the responsibility for raising CC awareness:

*They need to be told about HPV in any clinic…or any medical facility they approach.* Em 2

## Discussion

### Perspectives on cervical cancer and their influence on screening uptake

Some of the views of educated Muslim women interviewed for this study on the causes of CC were flawed. These included considering poor hygiene a cause, and particularly the use of public toilets, which was commonly believed to be a major causative factor in CC, as Wong et al. [13] also found. Findings of erroneous beliefs regarding CC causality of CC among educated women in this study are noteworthy. They suggest that awareness regarding CC and screening is low, even among educated women in the UAE. Respective studies by Al-Meer et al. conducted in Qatar and by Abdullah, Aziz and Su in Malaysia have also shown that formally educated women lack adequate knowledge of CC [16, 43]. An absence of symptoms led some women in this study to believe that they were free of CC, even though they were aware that CC was a ‘silent disease’. This finding lends minimal support to a study by Gan and Dahlui conducted in Malaysia that has suggested that screening increased as knowledge of symptoms increased [44]. In this study, some participants who were knowledgeable regarding CC and screening also did not have regular screening owing to their perception of low vulnerability.

Women’s knowledge of signs of CC was not always accurate (lumps in the vagina, uterus or external genitalia, loss of bowel control and loss of hair). However, most women correctly speculated that signs could include abnormal bleeding, pain and itching. Many women had been unaware of CC and screening until their doctors recommended screening. This finding is significant because it demonstrates that women are more likely to go for screening when a person deemed knowledgeable recommends that they do so. It also underlines the importance of involving all healthcare professionals in contact with female patients in fostering awareness on the need for CC screening. Wong et al. and El-Hammasi et al. have also respectively highlighted insufficient awareness in this area among women [8, 13]. These findings counter those of a quantitative study conducted in Sharjah, which found that most female teachers remained unscreened despite being knowledgeable regarding CC, thereby indicating that general awareness alone was insufficient for action [18]. A more individualistic approach is thus required to improve uptake of CC screening. However, participants in this study did not consider treatment and diagnosis futile. This finding contrasts with the findings of Wong et al. that diagnosis was considered pointless and that death was inevitable [13].

In this study, explicit questions related to personal sexual behaviour were not posed to respondents. However, unlike other studies, this study included perspectives on sexual relations, promiscuity and polygamy that were offered by participants of their own volition. Most participants felt that the risk of CC primarily increased as a result of extramarital relations, while a ‘good’ marriage could be protective against CC, thus lessening their own perceived risk. Polygamous marriages were neither considered promiscuous nor as a “bad relation” by the majority of the women interviewed. Thus, they were not associated with an increased risk of contracting CC. However, some expatriate women considered polygamy to be less acceptable as they associated lawful polygamy with an increased risk of CC. This finding suggests cultural variance in beliefs among Muslim women from different countries, necessitating awareness programmes that are designed to accommodate these differences. Promiscuity among Muslims was assumed to be less common than it was among non-Muslims. Women possibly avoided CC screening to maintain the image of a “successful” marriage, as screening could imply that their spouses had been unfaithful. However, many participants lacked the awareness to link CC to promiscuity. This study revealed the complexity of views within cultures and societies regarding tolerance of men’s promiscuity. Women faced more scrutiny by society and were held responsible for failed marriages, even when their husbands had been unfaithful. Consequently, they may have resisted screening to avoid ostracism within society.

### Perspectives on cervical cancer screening and their influence on screening uptake

Only three participants in this study described their screening experience as painful. This finding contrasts with that of a study in Sharjah where 42 % of the women participants perceived the screening process to be painful [27]. None of these participants revealed underlying conditions or previous procedures that could explain why they felt pain. Our study identified fear, embarrassment and pain felt by those who had never been screened before, or whose previous experiences had been painful, as barriers to screening. These findings are similar to those of earlier studies [8, 16, 18, 21, 45]. It is imperative that promotion of CC screening succeeds in allaying these fears to improve its uptake. It was apparent in our study that embarrassment was more of a concern among expatriates than among Emirati women, once again highlighting cultural differences.

The Islamic belief in the sanctity of life was emphasised by participants in this study. Women were certain that exposing their private parts, even to male physicians, was not prohibited in Islam if this was for the sake of health. This finding can be invaluable when promoting screening among Muslim women. Participants believed in the “evil eye”, while also emphasising that Islamic teaching advocated the pursuit of treatment, along with prayers to attain guidance and respite from God. Islam does not discourage women from seeking healthcare pertaining to parts of their body that they are expected to keep covered. The Muslim women in this study did not deem CC as a punishment inflicted by God, as suggested by Padela et al., but agreed that God had ultimate control over life and death, as also illustrated in previous qualitative studies on Muslim women [14, 15]. This finding is contrary to the implication drawn by a Malaysian study that low screening rates among Muslims could be attributed to religious barriers [12]. The current qualitative study offers a deeper understanding of the intricate religious beliefs of Muslim women and their impacts on women’s health behaviours. Women believed that reservations towards screening stemmed from cultural rather than religious beliefs and values.

To illustrate this point, the study elucidated that women felt an obligation, culturally, to think about others first. Consequently, they neglected their own health, particularly when they were asymptomatic or while considering preventative healthcare. Salman (2012) has also highlighted the belief held by Arab women that they were healthy unless symptomatic [15].

Further, Emirati women showed a preference for being accompanied by a family member while visiting a doctor, reflecting a cultural requisite of social acceptance. While approval from their husbands was desirable, women did not consider this a barrier to screening. This contributes to an understanding of the finding of the Sharjah study that 5.5 % of the women did not go for screening because of their husbands’ refusal of permission to be screened [18]. It also accentuates the value of cultivating social support among Muslim women and the need for health workers to strengthen acceptance and awareness of CC screening among all members of the society, including men [44, 46].

### Other factors influencing cervical cancer screening uptake

Muslim women stated their religious and personal preferences for female doctors during screening. This finding is common to other studies conducted in the Middle East and among Muslim women in London, respectively [8, 16, 21]. However, some Emirati women in our study indicated that they had greater faith in the competence of male doctors. This is another possible cultural influence. Some participants also indicated partiality towards Muslim doctors. However, in general, religion was not a major consideration when seeking a doctor. Many expatriates questioned the competence of Arab doctors. However, no clear preference was expressed for any particular nationality in relation to doctors. Being able to communicate with qualified, compassionate doctors in their preferred language was of paramount importance to participants. Other studies have not explored the influence of the nationality and religion of the doctor in relation to screening uptake.

While participants were content with screening facilities in Dubai, their general dissatisfaction with allopathic medicine has not been previously highlighted. Given perceptions that healthcare is becoming increasingly commercialised, alternative medicines are gaining in popularity, casting a negative light on screening. There is a need to emphasise the importance of patients’ satisfaction during interactions with health providers to restore their faith in allopathic medicine.

While health insurance is available to most Dubai residents, the cost has become a barrier to screening in cases where preferred healthcare providers or particular procedures are not covered by health insurance. Lack of widespread insurance coverage for preventative healthcare could prove detrimental to efforts to promote CC screening in the UAE. While Emiratis can avail free screening facilities in government hospitals, expatriates do not share these privileges. However, despite having access to free facilities, Emirati women in this study were not keen to get screened in government hospitals because of the long waiting times and their inability to choose preferred doctors for the procedure. This suggests the importance of enhancing cost-effective, efficient and patient-friendly services for preventative healthcare. Similar to the views expressed in Matin and Lebaron’s study [45], most women in our study would not consider CC screening to be a priority if they had to pay for it themselves.

### Awareness of cervical cancer, screening and future needs

A general lack of awareness among women regarding CC and screening is an important finding of this study, and has also been reported by other studies conducted in the Middle East [8, 17]. Gynaecologists provided most of the screening advocacy, as has also been revealed by a study conducted in Kuwait [18]. However, most Muslim women in this study felt that it was culturally appropriate to visit gynaecologists only after they were married or expecting a child. Sexually active unmarried women would find it difficult to seek CC screening because of these cultural expectations. Single women intending to get married in the UAE have access to reproductive health counselling when they undergo premarital testing to screen for genetic diseases. However, this does not include counselling on STDs or CC screening. Premarital testing is an established, culturally accepted health initiative that presents an enormous opportunity for health workers to target and educate women regarding CC screening.

Friends and families were a common source of information for women in this study. This was also found by Al Meer et al. [16] and highlights the importance of social interactions in disseminating information among Muslim women. Women health workers from within the community could play an invaluable role in reaching out to women who are culturally inclined to accept information disseminated by their own people. A preference to search the internet was seen among educated women when they sought information for themselves. Advertising reliable sources of electronic information as part of public health awareness initiatives targeting those who seek information themselves (for the sake of convenience or to avoid embarrassment) is essential to ensure the success of efforts to promote CC screening. Women emphasised the inadequacy of CC awareness programmes in Dubai. Once they became aware of the importance and potential benefits of CC screening in relation to the risk of getting CC, and its rising rate, most women were conscientious about being screened regularly.

## Limitations

This study has provided valuable insights into perspectives on CC among educated Muslim women of Dubai. However, it was limited to Emirati and South Asian women. Including expatriate Muslim women from other countries would have enabled a wider exploration of different cultural perspectives regarding CC screening. Inclusion of non-Muslim women residing in Dubai could also have deepened understanding of cultural influences in association with religious influences on women’s perspectives regarding CC screening.

## Implications for public health practice

The prevalence of CC is increasing in the UAE. This situation indicates the importance of an exploration of perspectives on CC and screening among Muslim women in Dubai for public health [3, 7].

Although most participants in our study had been screened, they remained unsure about the symptoms of CC. This finding is of particular relevance to public health because it indicates that awareness of symptoms and causes of CC are not necessarily a cue to take action, namely through screening. As suggested by the Health Belief Model, perceived susceptibility and severity are more likely to promote screening uptake than knowledge of the transmission and symptoms of CC which may require a more individualised approach [47, 48].

It was evident from this study that women learned about CC and screening only after marriage and when planning to have children. However, women are most likely to be infected with HPV soon after becoming sexually active [1]. They, therefore, need to develop awareness earlier through the initiation of a national awareness campaign. Such a campaign should be aimed at enlightening Muslin women on their susceptibility to CC and its rising trends in the UAE, understanding the benefits of screening and removing perceived cultural barriers towards screening [47]. CC education should also be incorporated into existing governmental-funded programmes.

All participants who had been screened had undergone the procedure on their gynaecologists’ recommendations, even when they themselves did not have a good understanding of what screening entailed. Thus, they put their faith in a trusted authority figure [47, 49]. This finding has enormous implications for public health. Not only does it indicate an avenue for further promotion of CC, but it also highlights the possibility that women who do not visit gynaecologists are less likely to be screened. This finding, therefore, emphasises the need to expand advocacy of opportunistic CC screening beyond just gynaecologists to all healthcare providers.

## Conclusions

The current study suggests that awareness regarding CC screening is inadequate among Dubai’s Muslim women. It has also shown that women are eager to learn more about the disease, indicating that there is fertile ground for promotion of CC screening within public health. However, changes in existing attitudes towards and within healthcare services are imperative to address sociocultural concerns of modesty, and men within this society also need to be educated on the importance of CC screening. Including screening as part of basic health insurance packages could help to raise CC screening rates. Because Muslim women often rely on community members for information, it is evident that community members could play an equally important role to that of health workers in disseminating information.

Cervical cancer screening among Muslim women is an under-researched subject, particularly in the Middle East where no qualitative study on CC screening has been conducted to date. This study makes a valuable contribution to furthering understanding of the perspectives of educated Muslim women in Dubai, especially in the context of their cultures, religion and sexual behaviours. It is evident from the study that weaknesses exist in the current opportunistic screening initiatives, thereby highlighting the need for a comprehensive national CC screening programme in Dubai. The potential success of such a programme hinges on incorporating the sociocultural and religious perspectives of educated Muslim women in Dubai into health care practices and the implementation of policies.

## Abbreviations

CC: Cervical cancer
Em: Emirati
Ex: Expatriate
HAAD: Health Authority Abu Dhabi
HPV: Human Papillomavirus
STD: Sexually transmitted disease
UAE: United Arab Emirates
WHO: Word Health Organisation
